# Risk Factors for Rebleeding after Emergency Endoscopic Treatment of Dieulafoy Lesion

**DOI:** 10.1155/2020/2385214

**Published:** 2020-08-24

**Authors:** Yongkang Lai, Jianfang Rong, Zhenhua Zhu, Wangdi Liao, Bimin Li, Yin Zhu, Youxiang Chen, Xu Shu

**Affiliations:** Department of Gastroenterology, The First Affiliated Hospital of Nanchang University, Nanchang, Jiangxi 330006, China

## Abstract

*Background and Objective*: Dieulafoy lesion is a rare, but life-threatening, cause of gastrointestinal hemorrhage, and endoscopic therapy is the preferred first-line treatment. The present study aims to analyze the risk factors for rebleeding after endoscopic hemostasis of gastroduodenal Dieulafoy lesion. *Methods*. A retrospective review of patients with Dieulafoy lesion who developed acute gastrointestinal bleeding and were treated primarily with endoscopic therapy from September 2014 to April 2019 was conducted. *Results*. A total of 133 patients with Dieulafoy lesion were included in the present study. The mean age of these patients was 56.05 ± 16.58 years, and 115 patients were male. Among these 133 patients, 26 patients developed rebleeding within 30 days of endoscopic therapy. The 30-day rebleeding rate for pure injection therapy (epinephrine, cyanoacrylate, or lauromacrogol injection alone), nonpure injection therapy (argon plasma coagulation, band ligation, and hemoclip application alone), and combination therapy (combination of any >2 methods) was 45.2%, 12.8%, and 11%, respectively. In the univariable analysis, endoscopic treatment, prothrombin time, gender, Rockall score, and leukocyte count were the risk factors for rebleeding. In the multivariable analysis, pure injection endoscopic treatment, white blood cells (>10 × 10^9^/L), and prothrombin time >12 seconds were the independent risk factors for rebleeding. *Conclusion*. Patients who undergo pure injection endoscopic treatment and have a high leukocyte count (>10 × 10^9^/L) or elevated prothrombin time (>12 seconds) have an increased risk of rebleeding within 30 days after endoscopic treatment for gastroduodenal Dieulafoy lesion. Combined endoscopic treatment is the most effective therapy to prevent rebleeding in gastroduodenal Dieulafoy lesion.

## 1. Introduction

Dieulafoy lesion (DL) is an infrequent cause of gastrointestinal (GI) bleeding, especially upper gastrointestinal (UGI) bleeding, and accounts for 1-2% of acute GI bleeding cases [1]. This disease was named after George Dieulafoy, who first described it in 1898 [2]. DL is a persistent, large, and tortuous artery, in which the diameter does not decrease when it reaches the mucosa from the submucosa [3]. The erosion of the mucosa and arterial wall leads to acute GI bleeding, which can be life-threatening [4]. DL predominantly occurs in the upper digestive tract, and approximately 95% of lesions occur in the stomach, especially in the gastric body [5]. DL can be found in the esophagus, ileum, jejunum, colon, rectum, and bronchus [6–9].

There are many ways to treat DL. Among these, endoscopic treatment remains as the preferred method due to the high success rate [10]. Various endoscopic techniques are available to achieve hemostasis. Barakat et al. reported that endoscopic band ligation and hemostatic clip are the most effective methods for DL [11]. Jiang et al. reported that cyanoacrylate injection and hemoclip placement are both effective in controlling bleeding from DL [12]. However, some patients can develop rebleeding after endoscopic therapy, which can also be life-threatening.

There are very few reports on the outcomes of combined endoscopic therapy for DL and its comparison with single endoscopic treatment. Furthermore, the reported studies have a small sample size. At present, there are no standard guidelines on the choice of endoscopic treatment for DL. In addition, the risk factors for the rebleeding of DL remain unknown. The present study aimed at investigating the risk factors for rebleeding within 30 days after emergency endoscopic treatment for DL and comparing the outcomes of different endoscopic hemostatic methods.

## 2. Materials and Methods

**Figure 1:**
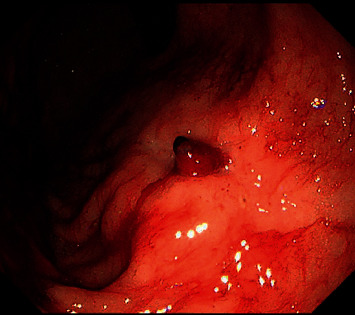
An endoscopic image showing a Dieulafoy lesion of approximately 0.5 cm in diameter near the lesser curve of the anterior wall of the body of the stomach.

**Figure 2:**
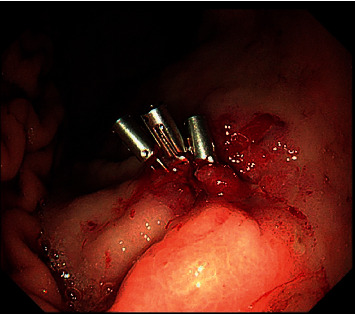
Endoscopic treatment by adrenaline plus cyanoacrylate injection around the lesion to stop the bleeding (10 ml at the 5 o'clock position) after hemoclip application.

**Figure 3:**
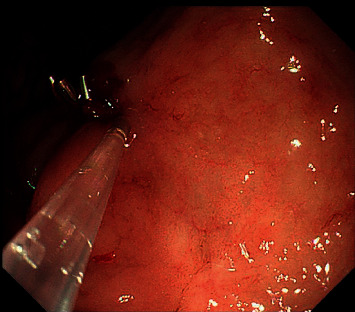
Endoscopic treatment of the Dieulafoy lesion by titanium hemoclip application.

**Figure 4:**
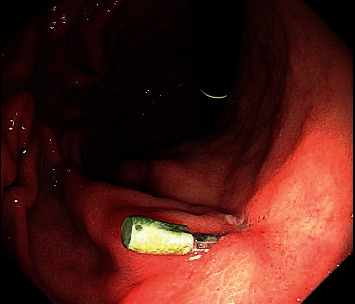
Endoscopic image of the residual clip, which can be observed at the site of 2 the original lesion with the resolution of the Dieulafoy lesion and no rebleeding.

In the present retrospective study, patients who underwent endoscopic treatment for acute GI bleeding due to DL at the Department of Gastroenterology, the First Affiliated Hospital of Nanchang University, China, from September 1, 2014, to April 11, 2019, were included. All emergency endoscopies were performed by an experienced deputy director or chief physician, and all included patients had follow-up records for at least 30 days (Figures 1–4). An ethics committee approval and written consent were not required for the present study.

### 2.1. Patient Selection

The present study included male or nonpregnant female patients within 15–90 years old, who were diagnosed to have DL in the gastroduodenal region on white light endoscopy and required hospitalization. The exclusion criteria were as follows: the diagnosis of DL could not be confirmed, DL was located in other parts of the gastrointestinal tract, and the presence of suspected malignancy.

### 2.2. Diagnosis of Dieulafoy Lesion

The endoscopic findings used for the diagnosis of DL were as follows [13]: (1) active arterial spurt, or pulsation from tiny mucosal defects or through the surrounding normal mucosa; (2) visualization of a protruding vessel, with or without active bleeding, within a minute mucosal defect or through the normal surrounding mucosa; and (3) the appearance of a fresh and densely adherent clot with a narrow point of attachment to a minute mucosal defect, or to a normal-appearing mucosa.

### 2.3. Data Collection

The collected data included the demographic information (age, gender, smoking, alcohol, comorbidities, and medication history), physical examination findings (blood pressure and heart rate), presenting symptoms, family history of UGI bleeding, laboratory reports, Forrest classification, AIMS65 score, Blatchford score, Rockall score, endoscopic treatment, and outcome (including death, rebleeding, and the need for surgery). A smoker was defined as a patient who smoked more than one cigarette per day for more than one year. Alcohol consumer was defined as patients who drank alcohol of any type for more than once every week, for at least one year. For the univariable and multivariable analysis, these patients were divided into three groups based on the endoscopic hemostatic method used: pure injection (including epinephrine injection plus cyanoacrylate injection and lauromacrogol injection alone), nonpure injection (including APC alone, band ligation alone, and hemoclip application alone), and combination therapy (any combination of two or three methods, as described above) (Table 1). All patients received intravenous proton pump inhibitors (80 mg and 8 mg/hour thereafter) for at least 72 hours postendoscopy.

The outcome measures included rebleeding rate, need for surgery, angiography, and mortality. Rebleeding was defined as recurrent hematemesis and melena with a fall in hemoglobin by at least 2 g/dL after the initial endoscopic treatment within 30 days. In the present study, rebleeding was used as the primary outcome to evaluate the risk factors.

### 2.4. Statistical Analysis

For the baseline characteristics, the variables were presented as mean ± standard deviation (SD) or proportion, as appropriate. The differences in baseline characteristics between the rebleeding and nonrebleeding groups were assessed using Student's *t*-test for continuous variables and chi-square test or Fisher's exact test for categorical variables, as appropriate. Univariable analysis was performed to assess the risk factors associated with rebleeding, and those with a *P* value of <0.20 were incorporated into the multivariable analysis. The results were presented as odds ratios (OR) with 95% confidence intervals (95% CI). *P* < 0.05 was considered statistically significant. The statistical analyses were performed using IBM SPSS Statistics for Windows (V.23.0).

## 3. Results

### 3.1. Patient Characteristics

**Figure 5:**
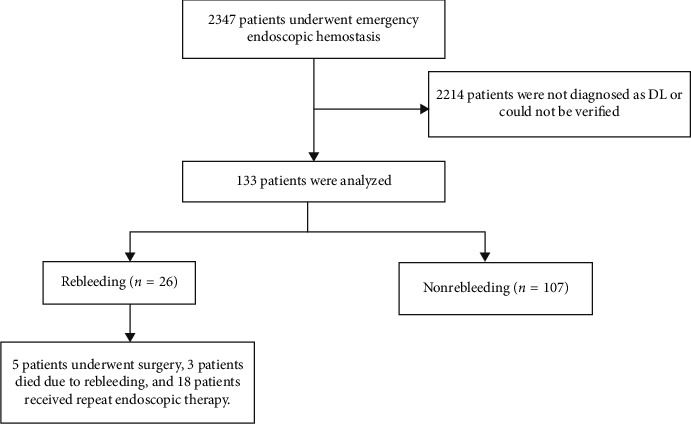
The flowchart of patients included in the present study.

A total of 2,347 patients had acute UGI bleeding and underwent an emergency endoscopy during the study period at our center. Among these patients, 133 patients (5.6%, Figure 5) with DL were included in the present study.

The mean age of these patients was 56.05 ± 16.58 years, and 115 (86.5%) of these patients were male. Rebleeding occurred in 26 (19.5%) patients, among which five (3.8%) patients required surgery. Three (2.3%) patients died due to rebleeding within 30 days. The most common site for DL was the gastric body (48.9%), followed by the duodenum (23.4%). A total of 96 (72.2%) patients had only DL on the endoscopy. Other lesions apart from the DL found on the endoscopy included inflammatory lesions at the anastomotic site, gastroduodenal ulcers, and erosive gastritis, and these were observed in 5, 24, and 8 patients, respectively. All patients with gastric remnant underwent gastrectomy for benign lesions. Among the 133 DL patients, active bleeding focus and recent hemorrhagic focus were present in 64 and 69 patients, respectively. The most frequent primary endoscopic hemostatic method chosen by endoscopists was combination therapy (41.4%), followed by nonpure injection (35.3%). Hematemesis was the most common symptom. The comorbidities included hypertension, type 2 diabetes mellitus, coronary artery disease, and renal failure, with hypertension (23 (17.3%)) being the most frequent comorbidity. None of the patients had liver disease or cirrhosis. Among the 133 DL patients, two patients were antiplatelet drug users and three patients were taking anticoagulants. Furthermore, 42 (31.6%) patients had a history of smoking and 31 (23.3%) patients were alcohol consumers. In addition, 47 patients had hypotension on admission. Hemoglobin (Hgb) at presentation ranged within 34–169 g/L, with a mean of 78.45 ± 23.26 g/L. Low platelet (PLT) count at presentation was observed in 27 patients. Deranged coagulation profile was present in 31 patients at the time of admission. The baseline characteristics are presented in detail in Table 1.

A comparison of the baseline characteristics of patients among pure injection, nonpure injection, and combination therapy indicated that the difference in age, gender, Forrest classification, Rockall score, AIMS65 score, Blatchford score, mean systolic blood pressure, heart rate, heart rate, WBC count, PLT, BUN, Cr, albumin, PT, APTT, and INR in each group was not significant. This indicates that the condition of patients in each group was not significantly different (Table 2).

### 3.2. Comparison between the Rebleeding and Nonrebleeding Groups

There were no significant differences between the rebleeding and nonrebleeding groups in terms of age, gender, DL location, Forrest classification, Rockall score, AIMS65 score, Blatchford score, clinical presentation, comorbidities, systolic blood pressure, heart rate, Hgb, PLT, blood urea nitrogen (BUN), prothrombin time (PT), activated partial thromboplastin time (APTT), and international normalized ratio (INR). Patients in the rebleeding group had lower diastolic blood pressure on admission (61.35 ± 11.19 vs. 67.04 ± 13.22; *P*=0.048), lower mean systolic blood pressure on admission (75.2 ± 11.3 vs. 82.45 ± 13.94; *P*=0.040), and lower serum albumin (28.83 ± 4.21 vs. 31.66 ± 6.49; *P*=0.050). On the other hand, mean white blood cell (WBC) count (10.45 ± 6.08 vs. 8.22 ± 3.9; *P*=0.029) and shock index (0.86 ± 0.18 vs. 0.77 ± 0.19; *P*=0.040) were higher in the rebleeding group.

Table 3 presents the univariable analysis of factors that affect the risk of rebleeding. On the univariable analysis, the type of endoscopic treatment, gender, WBC count, Rockall score, and PT were significantly associated with rebleeding. However, age, DL location, Forrest classification, clinical presentation, alcohol, smoking, history of peptic ulcer, AIMS65 score, Blatchford score, PLT, Hgb, and Cr had no apparent significance. On the multivariable analysis, pure injection endoscopic treatment (OR: 0.38; 95% CI: 0.20–0.72; *P*=0.003), WBC ≥ 10 × 10^9^/L (OR: 3.11; 95% CI: 1.17–8.31; *P*=0.023), and PT > 12 seconds (OR: 2.70; 95% CI: 1.02–7.17; *P*=0.046) were the independent risk factors for rebleeding (Table 3). For patients with risk factors (PT > 12 or WBC ≥ 10 × 10^9^/L), the rebleeding rate was higher in the pure injection group than in the nonpure injection and combination therapy groups (42.1% vs. 20.7% vs. 12.5%; *P*=0.048). For patients without risk factors, the rebleeding rate was higher in the pure injection group (50% vs. 0% vs. 8.7%; *P*=0.001) (Table 4).

## 4. Discussion

**Figure 6:**
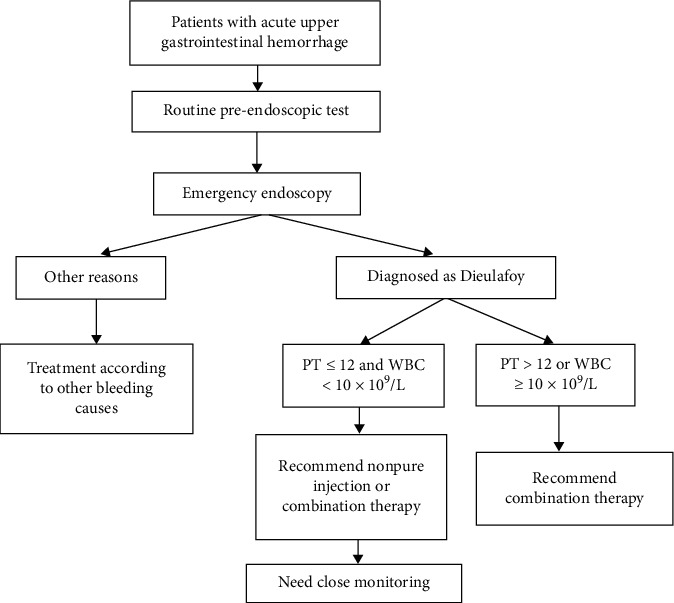
Treatment strategy according to risk factors.

Although DL is a not common cause of GI bleeding, a recent study revealed that DL accounts for 1.2%–4.0% of UGI bleeding cases [14, 15]. In the present study, it was found that patients who developed rebleeding had lower diastolic blood pressure, lower mean systolic blood pressure, and lower serum albumin on admission. In addition, patients with a higher WBC count and shock index appeared to have a higher risk of rebleeding within 30 days after endoscopic therapy. Furthermore, pure injection endoscopic treatment was an independent risk factor for DL rebleeding within 30 days, and the lowest risk of rebleeding was observed with the combined endoscopic therapy of DL. In addition, the present study revealed that the rebleeding rate was higher in the pure injection group, irrespective of the risk factors (Table 4). On the other hand, for patients without risk factors, the rebleeding rate was similar in the nonpure injection and combination therapy groups. Therefore, it is recommended that patients with high risk factors should be treated with combination therapy, while patients without high risk factors should be treated with noninjection or combination therapy (Figure 6).

Previous studies have revealed that DL is more frequent in elderly people (>60 years old) [3, 16]. However, in the present study, 73 patients with DL were <60 years old. This finding could be due to the small sample size of previous studies, or due to racial differences. In the present study, WBC ≥ 10 × 10^9^/L was one of the risk factors for DL rebleeding. WBC ≥ 10 × 10^9^/L was associated with almost 3-fold times higher risk of rebleeding, when compared to patients with WBC ≤ 10 × 10^9^/L on admission. Leukocytosis is a marker of infection and inflammation. Previous studies have revealed the significant correlation between elevated leukocyte count and mortality in patients with peptic ulcer [17, 18]. In addition, high leukocyte count also indicates serious illness [19]. In the present study, it was speculated that WBC ≥ 10 × 10^9^/L reflected the severity of DL bleeding, or revealed that the mucosal ulcer was infected and has a higher risk of hemorrhage. Hence, it may be imperative for clinicians to closely follow up DL patients with a high WBC count, since they have a higher risk of rebleeding. Elevated PT was identified as another independent risk factor for DL rebleeding. Prolonged PT indicates deranged coagulation function and could be the reason for the secondary rebleeding from the site of endoscopic clipping or injection therapy [20]. Hence, for such patients, and along with endoscopic therapy, the coagulation profile should be corrected by stopping the anticoagulants, especially when the patient is taking any of these, and administering fresh frozen plasma. In addition, for patients with prolonged PT and increased leukocyte count, combined endoscopic therapy should be preferred. Although other ulcers were found during the endoscopy, since none of these other ulcers had bleeding manifestations, it was not considered that the rebleeding after the endoscopic hemostasis was caused by ulcers in other parts.

The previous study conducted by Ding et al. revealed that hemoclip application is a safe and effective treatment for preventing DL rebleeding [16]. Alis et al. reported that bang ligation was superior to other endoscopic methods for preventing rebleeding in DL [21]. Iacopini et al. reported that thermal coagulation is an effective endoscopic method [22]. Kanth et al. and Sone et al. reported that combined endoscopic therapy may be the best way to reduce rebleeding [3, 14]. There were also several other studies, in which various endoscopic methods have been successfully used to treat DL [23–25]. However, since most of these studies had a small sample size, a multivariable regression analysis was not performed, and the independent risk factors for rebleeding could not be appropriately identified.

There were some limitations of the present study. First, the present study was a single-center retrospective study. Hence, the findings of the present study need to be validated through multicenter prospective studies. Second, electrocoagulation hemostasis was not separately identified as a type of noninjection endoscopic treatment, and tissue gel injection was also not separated from the cyanoacrylate injection, which may lead to errors in the results. Third, the present study only reported the outcomes of the endoscopic treatment of DL in the gastroduodenal region, and these could not be extrapolated to the DL at the other sites. Fourth, the experience and proficiency of different endoscopists may have had an impact on the results.

In conclusion, the pure injection type of endoscopic treatment, elevated leukocyte count (>10 × 10^8^/L), and elevated prothrombin time (>12 seconds) were the independent risk factors for rebleeding within 30 days after the endoscopic treatment of gastroduodenal DL. The early identification and treatment of these risk factors can help to prevent rebleeding. Combined endoscopic treatment should be the preferred choice, since this can effectively prevent rebleeding in gastroduodenal DL.

## Figures and Tables

**Table 1:** Baseline characteristics between the rebleeding and nonrebleeding group.

Characteristic		Rebleeding (*n* = 26)	Nonrebleeding (*n* = 107)	*P* value	Total (*n* = 133)
Age, years, mean ± SD		54.42 ± 19.26	56.45 ± 15.94	0.58	56.05 ± 16.58
	≤60 (%)	16 (21.9)	57 (78.1)	0.45	73 (54.9)
	>60 (%)	10 (16.7)	50 (83.3)		60 (45.1)

Gender	Male (%)	20 (76.9)	95 (88.8)	0.12	115 (86.5)
	Female (%)	6 (23.1)	12 (11.2)		18 (13.5)

DL location	Fundus (%)	2 (7.7)	12 (11.2)	0.45	14 (10.5)
	Body (%)	12 (46.3)	53 (49.5)		65 (48.9)
	Angulus (%)	1 (3.8)	3 (2.8)		4 (3)
	Antrum (%)	0	7 (6.5)		7 (5.3)
	Duodenum (%)	9 (34.6)	22 (20.6)		31 (23.4)
	Anastomotic site (%)	2 (7.6)	10 (9.3)		12 (9)

Other significant endoscopic findings	None	75 (70.1)	21(80.8)	0.82	96 (72.2)
	Other gastroduodenal ulcer	5 (4.7)	0		5 (3.8)
	Anastomositis	20 (18.7)	4(15.4)		24 (18)
	Erosive gastritis	7 (6.5)	1 (3.8)		8 (6)

Forrest classification	FI	14 (21.9)	50 (78.1)	0.52	64 (48.1)
	FII	12 (17.4)	57 (82.6)		69 (51.9)

Rockall score	Moderate risk group (score 3-4) (%)	18 (69.2)	90 (84.1)	0.09	108 (81.2)
	High risk group (score ≥5) (%)	8 (30.8)	17 (15.9)		25 (18.8)

AIMS65 score	<2 (%)	16 (17.2)	77 (82.8)	0.3	93 (69.9)
	≥2 (%)	10 (25)	30 (75)		40 (30.1)

Blatchford score	Low-risk group (score <6) (%)	1(3.8)	10(9.3)	0.38	11 (8.3)
	High-risk group (score ≥6) (%)	25 (96.2)	97 (90.7)		122 (91.7)

Endoscopy treatment	Pure injection (%)	14 (45.2)	17 (54.8)	0.02	31 (23.3)
	Nonpure injection (%)	6 (12.8)	41 (87.2)		47 (35.3)
	Combination therapy (%)	6 (11)	49 (89)		55 (41.4)

Clinical presentation	Hematemesis (%)	20 (22)	71 (78)	0.35	91 (68.4)
	Melena (%)	6 (14.3)	36 (85.7)		42 (31.6)

Alcohol (%)		4 (12.9)	27 (87.1)	0.29	31 (23.3)
Smoking (%)		9 (34.6)	33 (30.8)	0.72	42 (31.6)
NSAIDs (%)		0	2 (1.9)	1	2 (1.5)
Anticoagulants (%)		0	2 (1.9)	1	2 (1.5)
History of peptic ulcer (%)		4 (15.4)	15 (14)	1	19 (14.3)
Hypertension (%)		2 (7.7)	20 (19.6)	0.24	22 (17.3)
Diabetes mellitus (%)		0	6 (5.6)	0.6	6 (4.5)
Coronary artery disease (%)		0	3 (2.8)	1	3 (2.3)
Renal failure (%)		1(3.8)	0	0.36	1 (0.8)
Systolic blood pressure, mean ± SD		105.88 ± 17.67	113.29 ± 18.6	0.07	111.84 ± 18.59
Diastolic blood pressure, mean ± SD		61.35 ± 11.19	67.04 ± 13.22	0.05	65.92 ± 13
Mean systolic blood pressure, mean ± SD		75.2 ± 11.3	82.45 ± 13.94	0.04	81.23 ± 13.77
Heart rate, mean ± SD		88.92 ± 18.15	85.31 ± 18.9	0.38	86.02 ± 18.74
Shock index, mean ± SD		0.86 ± 0.18	0.77 ± 0.19	0.04	0.79 ± 0.2
Hemoglobin, mean ± SD		74.73 ± 15.66	79.37 ± 24.73	0.36	78.45 ± 23.26
WBC (109/L), mean ± SD		10.45 ± 6.08	8.22 ± 3.9	0.03	8.66 ± 4.47
PLT (109/L), mean ± SD		158.31 ± 63.41	174.81 ± 107.41	0.45	171.56 ± 100.34
BUN (mmol/L), mean ± SD		9.59 ± 7.14	9.86 ± 6.57	0.84	9.8 ± 6.66
Cr (umol/L), mean ± SD		103.16 ± 158.1	80.77 ± 25.83	0.26	85.22 ± 73.12
Albumin (g/L), mean ± SD		28.83 ± 4.21	31.66 ± 6.49	0.05	31.09 ± 6.2
PT (s), mean ± SD		12.99 ± 2.58	13.26 ± 9.78	0.89	13.2 ± 8.84
APTT (s), mean ± SD		33.7 ± 9.33	29.61 ± 10.85	0.11	30.41 ± 10.66
INR, mean ± SD		1.15 ± 0.24	1.08 ± 0.17	0.11	1.1 ± 0.19
Operation (%)		5 (19.2)	0	0.001	5 (3.8)
Mortality (%)		3 (11.5)	0	0.05	3 (2.3)

**Table 2:** Comparison of baseline clinical characteristics among pure injection, nonpure injection, and combination therapy.

Characteristic	Pure injection (*n* = 31)	Nonpure injection (*n* = 47)	Combination therapy (*n* = 55)	*P* value
Age, years, mean ± SD	56.52 ± 3.29	55 ± 2.62	56.69 ± 1.95	0.865
Gender, male	23 (74.19%)	42 (89.36%)	50 (90.91%)	0.096
DL location, stomach	15 (48.39%)	35 (74.47%)	40 (72.73%)	0.032
Forrest classification, FI	14 (45.16%)	20 (42.55%)	30 (54.55%)	0.449
Rockall score	3.84 ± 0.19	3.91 ± 0.16	3.76 ± 0.12	0.745
AIMS65 score	0.97 ± 0.16	1.02 ± 0.15	1.05 ± 0.12	0.921
Blatchford score	9.03 ± 0.51	9.28 ± 0.46	8.95 ± 0.36	0.841
Alcohol	3 (9.68%)	10 (21.28%)	18 (32.73%)	0.048
Smoking	9 (29.03%)	18 (38.3%)	15 (27.27%)	0.461
History of peptic ulcer	2 (6.45%)	9 (19.15%)	8 (14.55%)	0.274
Hypertension	6 (19.35%)	11 (23.4%)	5 (9.1%)	0.136
Diabetes mellitus	0	3 (6.38%)	3 (5.45%)	0.498
Mean systolic blood pressure, mean ± SD	78.55 ± 2.09	80.74 ± 2.14	83.13 ± 1.9	0.324
Heart rate, mean ± SD	89.39 ± 3.07	88.19 ± 2.91	81.75 ± 2.43	0.086
Hemoglobin, mean ± SD	77.16 ± 3.91	79.49 ± 3.36	78.29 ± 3.32	0.91
WBC (10^9^/L), mean ± SD	8.67 ± 0.82	9.23 ± 0.71	8.16 ± 0.55	0.488
PLT (10^9^/L), mean ± SD	182.94 ± 12.44	173.84 ± 14.52	163.215.59	0.672
BUN (mmol/L), mean ± SD	10.09 ± 1.29	9.3 ± 0.64	10.07 ± 1.07	0.814
Cr (umol/L), mean ± SD	102.39 ± 26.14	77.68 ± 3.51	82.11 ± 3.21	0.517
Albumin (g/L), mean ± SD	30.77 ± 1.03	32.28 ± 0.98	30.31 ± 0.79	0.261
PT (s), mean ± SD	12.1 ± 0.3	12.58 ± 0.33	14.37 ± 1.82	0.434
APTT (s), mean ± SD	30.16 ± 1.09	30.94 ± 1.82	30.16 ± 1.49	0.924
INR, mean ± SD	1.06 ± 0.03	1.11 ± 0.03	1.11 ± 0.03	0.447
Operation	4 (12.9%)	1 (2.13%)	0	0.005
Mortality	1(3.23%)	1(2.13%)	1(1.82%)	1

**Table 3:** Univariable and multivariable analysis for rebleeding as the primary outcome.

Variable	Univariate OR (95% CI)	*P* value	Multivariate OR (95% CI)	*P* value
Age >60	0.71 (0.30–1.71)	0.45		
Gender	0.42 (0.14–1.26)	0.12	0.56 (0.16–2.02)	0.376
DL location	1.72 (0.71–4.15)	0.23		
Forrest classification	0.75 (0.32–1.78)	0.52		
Endoscopic treatment	0.36 (0.20–0.66)	0.001	0.38 (0.20–0.72)	0.003
Clinical presentation	0.59 (0.22–1.6)	0.3		
Alcohol	0.54 (0.17–1.70)	0.29		
Smoking	1.19 (0.48–2.94)	0.71		
History of peptic ulcer	1.12 (0.34–3.69)	0.86		
Hypertension	0.36 (0.08–1.66)	0.21		
Rockall score, high-risk group (score ≥5)	2.35 (0.99–6.26)	0.09	1.87 (0.64–5.52)	0.255
AIMS65 score, ≥2	1.6 (0.66–3.93)	0.3		
Blatchford score, high-risk group (score ≥6)	2.58 (0.32–21.09)	0.38		
WBC ≥10×109/L	2.86 (1.19–6.87)	0.02	3.11 (1.17–8.31)	0.023
PLT ≥150	1.48 (0.61–3.61)	0.39		
HGB ≥100	0.51 (0.11–2.40)	0.4		
Cr > 106	1.27 (0.32–4.97)	0.74		
PT > 12	2.38 (0.99–5.74)	0.05	2.70 (1.02–7.17)	0.046

**Table 4:** Rebleeding rate of each hemostatic technique based on the risk factors.

		Pure injection	Nonpure injection	Combination therapy	*P*
PT > 12 or WBC ≥10 × 10^9^/L	Rebleeding	8(42.1%)	6(20.7%)	4(12.5%)	0.048
	Nonrebleeding	11(57.9%)	23(79.3%)	28(87.5%)	

PT ≤ 12 and WBC <10 × 10^9^/L	Rebleeding	6(50%)	0	2(8.7%)	0.001
	Nonrebleeding	6(50%)	18	21(91.3%)	

## Data Availability

The clinical data were not made public to protect the privacy of the patients. However, the data can be made available upon reasonable request by email.
